# The bromo-adjacent homology domains of PBRM1 associate with histone tails and contribute to PBAF-mediated gene regulation

**DOI:** 10.1016/j.jbc.2023.104996

**Published:** 2023-06-30

**Authors:** Christopher J. Petell, Nathaniel T. Burkholder, Paloma A. Ruiz, Jessica Skela, Jake R. Foreman, Lauren E. Southwell, Brenda R. Temple, Krzysztof Krajewski, Brian D. Strahl

**Affiliations:** 1 Department of Biochemistry and Biophysics, University of North Carolina at Chapel Hill, Chapel Hill, North Carolina, USA; 2 UNC Lineberger Comprehensive Cancer Center, University of North Carolina at Chapel Hill, Chapel Hill, North Carolina, USA; 3 R L Juliano Structural Bioinformatics Core Facility, University of North Carolina at Chapel Hill, Chapel Hill, North Carolina, USA

**Keywords:** PBRM1, BAF180, SWI/SNF complex, histones, chromatin, chromatin regulation, epigenetics, Transcription

## Abstract

A critical component of gene regulation is recognition of histones and their post-translational modifications by transcription-associated proteins or complexes. Although many histone-binding reader modules have been characterized, the bromo-adjacent homology (BAH) domain family of readers is still poorly characterized. A pre-eminent member of this family is PBRM1 (BAF180), a component of the PBAF chromatin-remodeling complex. PBRM1 contains two adjacent BAH domains of unknown histone-binding potential. We evaluated the tandem BAH domains for their capacity to associate with histones and to contribute to PBAF-mediated gene regulation. The BAH1 and BAH2 domains of human PBRM1 broadly interacted with histone tails, but they showed a preference for unmodified N-termini of histones H3 and H4. Molecular modeling and comparison of the BAH1 and BAH2 domains with other BAH readers pointed to a conserved binding mode *via* an extended open pocket and, in general, an aromatic cage for histone lysine binding. Point mutants that were predicted to disrupt the interaction between the BAH domains and histones reduced histone binding *in vitro* and resulted in dysregulation of genes targeted by PBAF *in cellulo*. Although the BAH domains in PBRM1 were important for PBAF-mediated gene regulation, we found that overall chromatin targeting of PBRM1 was not dependent on BAH-histone interaction. Our findings identify a function of the PBRM1 BAH domains in PBAF activity that is likely mediated by histone tail interaction.

Eukaryotic DNA is packaged into chromatin, which consists of DNA wrapped around histones and associated non-histone proteins (1, 2). The histones are modified by a large assortment of post-translational modifications (PTMs) that regulate chromatin function; PTMs include methylation, acetylation, phosphorylation, and ubiquitination. Although these PTMs can biophysically impact the charged interactions between histone tails and DNA, they also function by recruiting various transcription factors and other chromatin-binding proteins (3, 4). Histone modifications have been linked to different transcriptional states and histone modifications affect the activity of transcriptional complexes, thereby influencing gene expression profiles and cell identity (5, 6). The deposition, removal, and interpretation (*i.e.*, reading) of these histone modifications are performed by many epigenetic effectors that employ a diverse set of possible reader modules that detect specific PTMs (7, 8, 9, 10).

An emphasis in the chromatin and epigenetics fields is the characterization of understudied families of putative histone reader domains. Importantly, cancer and other diseases are often associated with overexpression or mutation of histone PTM reader proteins (11, 12, 13). The bromo-adjacent homology (BAH) domain family is one such family that has been understudied but is of great interest to epigenetics researchers. In humans, the BAH family includes factors that have important functions in replication (ORC1), DNA methylation (DNMT1 and ZMET2), and nucleosome remodeling (PBRM1) (14, 15). Interestingly, BAH domains thus far have been shown to have a variety of histone-binding preferences that include the nucleosome core particle and unmodified H3K79 (Sir3), H4K20me2 (ORC1), H3K9me3 (ZMET2), and H3K27me3 (BAHD1) (16, 17, 18, 19, 20, 21). In many cases, the loss of the aforementioned histone-reading activity leads to defects in the overall functions of the histone-reader-containing complexes.

PBRM1 is a subunit of the PBAF nucleosome-remodeling complex, which has functions in regulating transcription and facilitating DNA repair (22). PBRM1 is expressed in all tissues examined to date and is functionally conserved among eukaryotes (23, 24). PBRM1 contains a high-mobility group (HMG) DNA-binding domain, two uncharacterized BAH domains, and six bromodomains (BRDs) (Fig. 1*A*). BRD2 and BRD4 associate with H3K14ac and function in maintaining PBAF on acetylated chromatin (25). PBRM1 is mutated in ∼4% of all known cancers and ∼40% of all clear cell renal cell carcinomas (ccRCC), whereas other subunits of PBAF are also frequently mutated in cancer (26, 27, 28, 29, 30). Despite the importance of other BAH domains in histone-mediated regulation, it is not known how the BAH domains of PBRM1 contribute to PBAF function in mammals. However, studies of the yeast PBRM1 orthologs Rsc1 and Rsc2 do support a requirement of the BAH domains and histone recognition in the function of the PBAF-orthologous RSC complex (31, 32, 33, 34, 35, 36).

**Figure 1:**
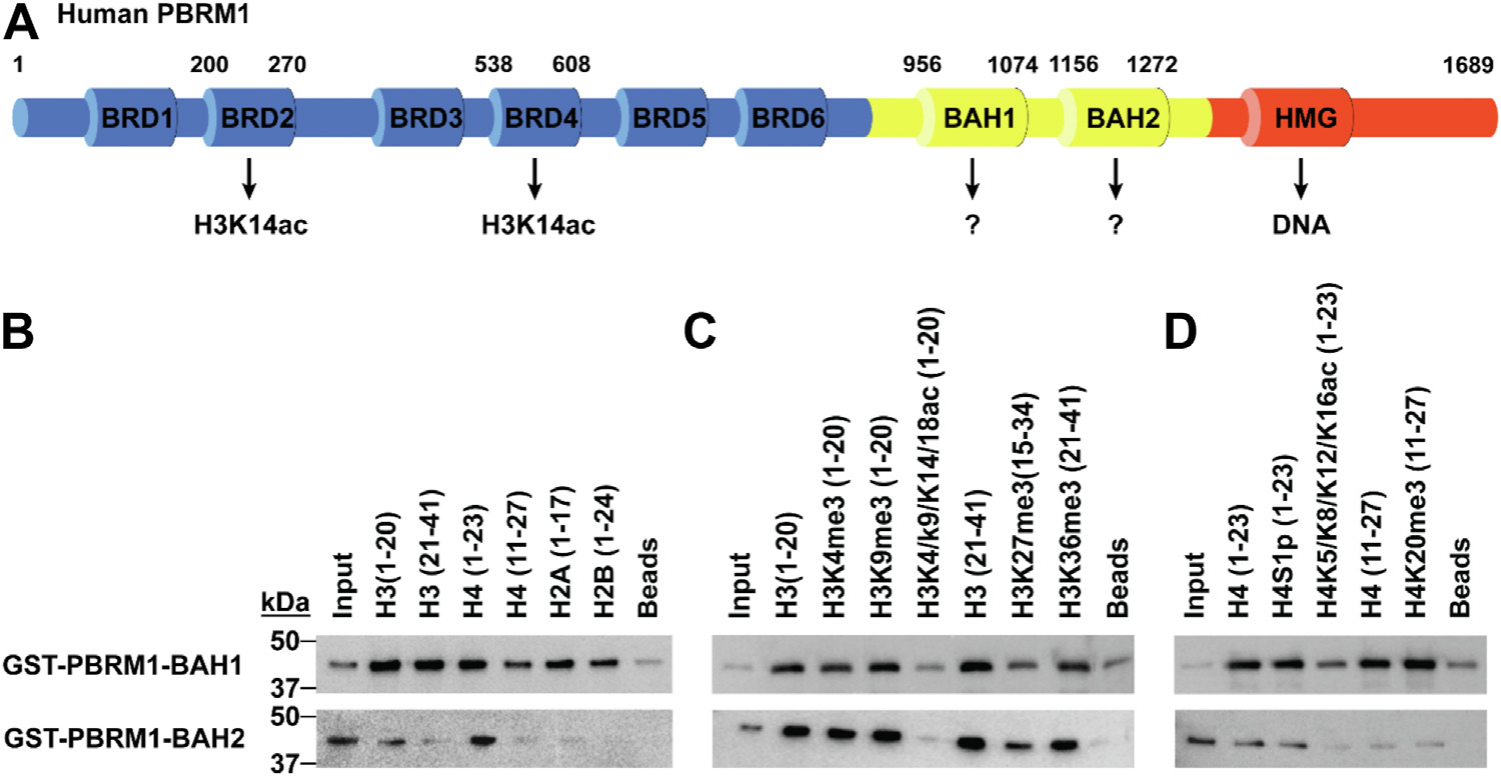
**The two BAH domains of human PBRM1 have histone binding activity *in vitro*.***A*, domain architecture of human PBRM1. Indicated are known interactions of the two bromodomains (BRD2/4, *blue*) and high-mobility group domain (HMG, *red*) with histones and DNA, respectively. Interactions for the two bromo-adjacent homology domains (BAH1 and BAH2, *yellow*) have not been characterized. *B*–*D*, histone peptide pulldown screens of purified GST-linked human PBRM1 BAH1 and BAH2 domains (∼42 kDa expected). A 1% input was included for loading validation and no peptide pulldown (Beads) was included as a negative control. Images shown are representative of n ≥ 3 experiments. *B*, peptide pulldowns walking across the unmodified canonical histone tails. *C*, peptide pulldowns with common H3 modification states. *D*, peptide pulldowns with common H4 modification states.

We, therefore, sought to determine if the BAH domains of PBRM1 are indeed histone readers and whether they contribute to PBRM1's function. We found that GST-fusions of human PBRM1 BAH1- and BAH2-bound histones and generally were selective for unmodified H3 and H4 N-termini with an aversion for poly-acetylation and trimethylation of H3K27. Structural homology modeling and comparison of the PBRM1 BAH1 and BAH2 domains with other characterized BAH domains revealed that the PBRM1 BAH1 and BAH2 have similar folds and share similar aromatic cages that are critical for histone binding by other BAH domains (20). Mutation of PBRM1 BAH1 and BAH2 residues that were predicted to be important for histone interaction as well as mutation of residues that occur in various cancers, reduced histone-BAH domain interactions *in vitro*. Knockdown and replacement of PBRM1 containing BAH-histone blocking mutations did not however significantly alter PBRM1’s recruitment to chromatin in a human cell model. Nevertheless, replacement with the BAH-mutated PBRM1 did significantly alter the expression of genes that are regulated by PBAF. These studies highlight an important function of the PBRM1 BAH domains in gene regulation and show that these domains can associate with histone tails.

## Results

### The PBRM1 BAH domains broadly associate with H3 and H4 histone N-termini in the absence of poly-acetylation

PBRM1 consists of a series of six bromodomains (BRDs), two bromo-adjacent homology (BAH) domains, and a high-mobility group (HMG) domain (Fig. 1*A*). Chambers *et. al.* reported that that BRD2 and BRD4 of PBRM1 recognized H3K14ac (37, 38). In contrast to this binding activity of the BRDs, little is known regarding the histone-binding preferences of the BAH domains of PBRM1.

To determine the histone or histone PTM preference(s) of the PBRM1 BAH domains, we expressed and purified the human PBRM1 BAH1 or BAH2 proteins as GST fusions in *E. coli*. Our initial attempts to screen these GST fusions on customized peptide microarrays (39) did not reveal any histone or histone PTM interactions (data not shown). However, domains with weak histone affinity (>20 μM) are poorly detected in this assay. We, therefore, performed solution-based peptide pulldown assays with a range of candidate histone peptides to examine the potential of the PBRM1 BAH domains to bind to histones or histone PTMs. Our initial peptide pulldowns revealed significant non-specific interactions of both BAH domains with the beads-only control in standard buffer conditions (data not shown). Thus, for these domains, we optimized the pulldown conditions by using more stringent binding conditions (see Experimental Procedures). Screening with several H3 and H4 peptides revealed abundant interactions for both PBRM1 BAH domains with various histone tails; the strongest histone binding was observed for the BAH1 domain (Fig. 1*B*). The BAH1 promiscuously bound to each of the canonical histone tail peptides, with a bias toward H3 and H4 N-terminal tails. In comparison, the BAH2 preferentially bound to both N-terminal H3 and H4 peptides with greater selectivity than for H2A and H2B N-termini (Fig. 1*B*).

Given that H3 and H4 tails were the predominant targets of the PBRM1 BAH1 and BAH2 domains, we next sought to determine how different PTMs impacted PBRM1 BAH binding to histones. Interestingly, both the BAH1 and BAH2 domains bound similarly to H3 unmodified peptides and many H3 modification states (Fig. 1*C*). There were exceptions to this rule, namely, reduced binding to H3K27me3 and greatly reduced binding to poly-acetylated peptides for both BAH domains (Fig. 1*C*). In the context of H4 modifications, we again found that most H4 modification states generally had minimal impact on BAH1 and BAH2 histone binding (Fig. 1*D*). Similar to the finding for H3 binding, we observed reduced binding of the BAH domains with poly-acetylated H4 peptides (Fig. 1*D*). As a whole, our analyses showed that both BAH domains of PBRM1 were competent histone reader domains that had reduced binding towards H3/H4 poly-acetylated and H3K27me3 modified histone tails.

### Structural homology modeling of the PBRM1 BAH1 and BAH2 domains point to a conserved histone-binding mode

**Figure 2:**
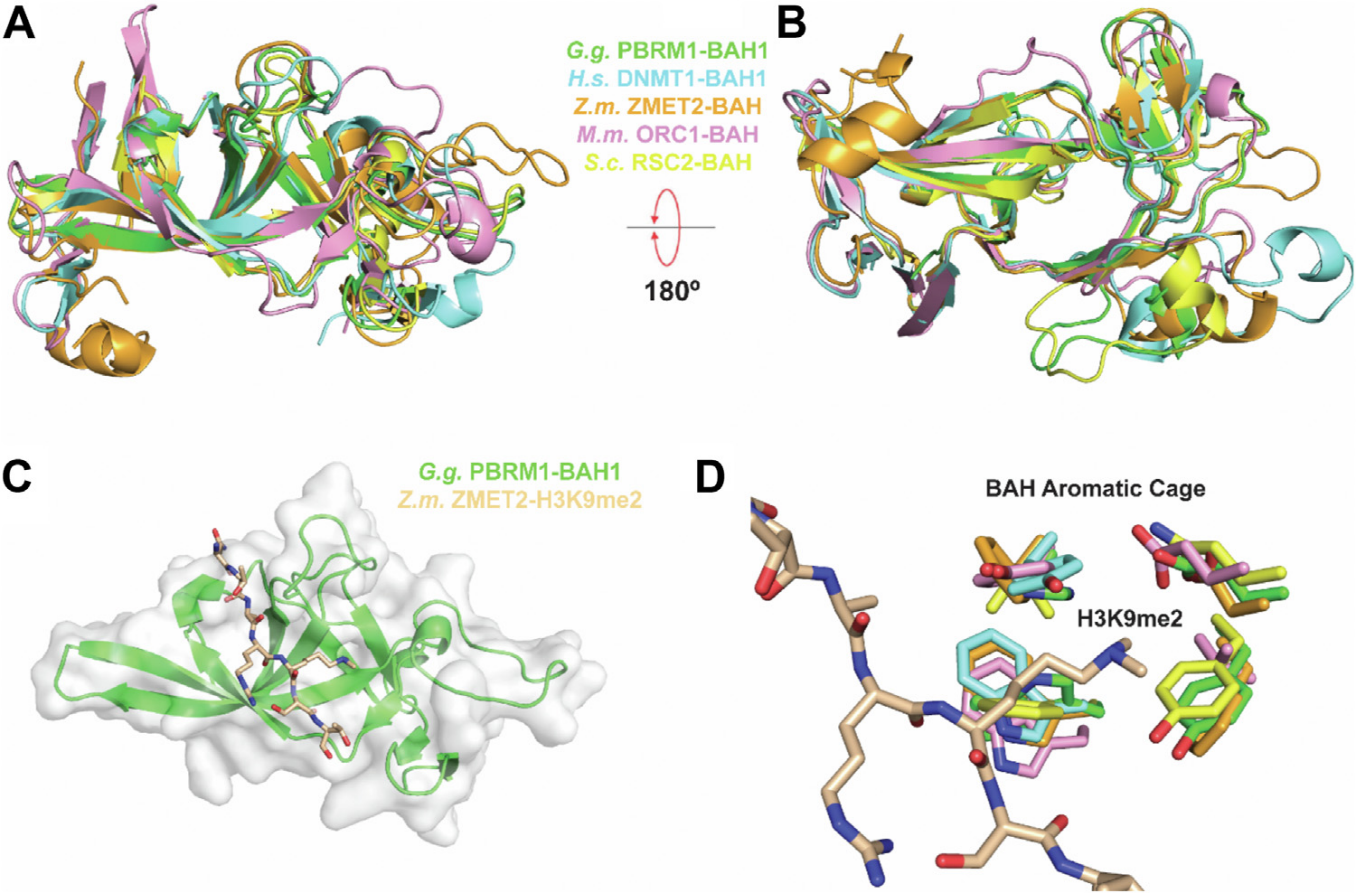
**Structural conservation of the BAH domain across distinct phyla points to a conserved histone-binding mechanism for the BAH domains of human PBRM1.***A* and *B*, structural alignment of the HHpred-identified homologous BAH domains from *Gallus gallus* PBRM1 (PDB:1W4S, *green*), *Homo sapiens* DNMT1 (PDB:3SWR, *light blue*), *Zea mays* ZMET2 (PDB:4FT4, *orange*), *Mus musculus* ORC1 (PDB:4DOV, *pink*), and *Saccharomyces cerevisiae* Rsc2 (PDB:4BB7, *yellow*). *B*, 180° rotation of alignment shown in *A*). *C*, H3K9me2-bound peptide (tan) from *Z.m.* ZMET2 structure aligned with *G.g.* PBRM1-BAH1 conserved binding pocket. *D*, conserved histone modification binding residues (all within 4 Å) from aligned BAH domains.

To further explain how the BAH domains of PBRM1 associate with histones, we next employed the structural prediction tool HHpred, which can examine the structures of closely related domains to generate a highly predictive model of an uncharacterized domain (40). In addition to predicting overall structure, HHpred also can predict conserved folds and binding pockets, such as those used by reader domains to bind to histones (41). To generate models of the PBRM1 BAH1 and BAH2 domains, we used HHpred to identify all potential structural templates in which the BAH domain was either highly conserved (*e.g.*, yeast RSC2 and chicken PBRM1) or in which a BAH domain structure had been solved in complex with a histone peptide (*i.e.*, *Zea mays* ZMET2 and *Arabidopsis* ORC1). Although the overall BAH sequences differ greatly (Fig. S1*A*), templates identified by HHpred revealed a conserved folding among the BAH domains examined (Fig. 2, *A* and *B*). Importantly, these conserved elements generally form a groove that appear to be able to accommodate a histone tail (Figs. 2*C* and S1*B*), potentially akin to that of the *Z. mays* ZMET2 BAH which binds to H3K9me2 (20). Additionally, we identified a conserved BAH aromatic cage for putative histone lysine binding based on our broad homology alignment (Fig. 2*D*). These observations suggest a conserved histone binding mode for the BAH domain family.

**Figure 3:**
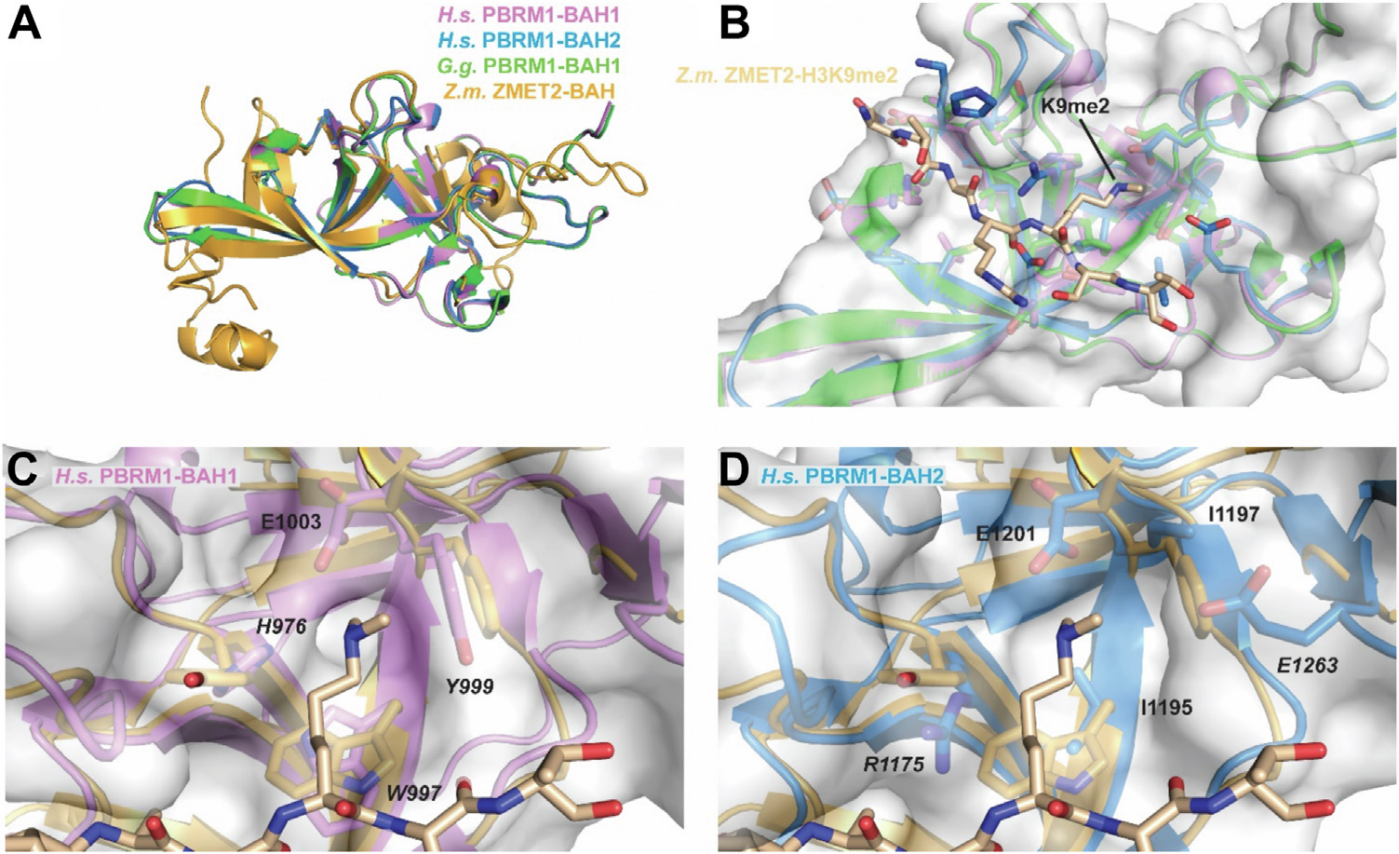
**Structural homology modeling of the human PBRM1 BAH domains illustrate how they may recognize histone modifications.***A*, structural alignment of the BAH domains from *Gallus gallus* PBRM1 (PDB:1W4S, *green*), *Zea mays* ZMET2 (PDB:4FT4, *orange*), and the *Homo sapiens* PBRM1 BAH1/2 homology models (BAH1, violet; BAH2, *blue*). *B*, alignment of *G.g.* and *H.s.* PBRM1 BAH domains with *Z.m.* ZMET2 H3K9me2-bound peptide (tan). *C* and *D*, residues that form aromatic and atypical cages of the PBRM1 BAH1 and BAH2 domains, respectively.

Although a publicly available structure of the chicken BAH1 domain was the closest template for the human BAH1 and BAH2 domains based on sequence alignment and structural prediction, we also aimed to generate models that predicted how the human BAH domains could bind histones. Thus, we used HHpred to thread the human PBRM1 BAH domains not only onto the *Gallus gallus* BAH1 structure but also onto the histone-bound *Z. mays* ZMET2 BAH structure for predictive modeling of human BAH histone peptide binding (Fig. 3*A*). This threading approach yielded models for the human PBRM1 BAH1 and BAH2 domains that appeared to have similar deep grooves capable of accommodating histone tails (Fig. 3*B*). The human PBRM1 BAH1 appears to have an intact aromatic cage consisting of residues H976, W997, and Y999 which were similar to the aromatic cage residues of the ZMET2 BAH (Fig. 3*C*). However, the PBRM1 BAH2 domain showed a relatively atypical BAH cage consisting of two small hydrophobic residues, I1195 and I1197, and a positively charged R1175 in place of the usual aromatic residues (Fig. 3*D*). The absence of aromatic residues likely explains the apparent weaker histone binding of the BAH2 compared to the BAH1 domain of PBRM1 (see Fig. 1*B*). We note that the presence of an auxiliary long negatively charged residue E1263 may stabilize histone lysine binding in the absence of an aromatic residue (Fig. 3*D*). Additionally, because of perturbation of histone binding by poly-acetylation (Fig. 1, *C* and *D*), we performed electrostatic potential mapping of our models and found that both BAH models have a shape and charge potential in the putative peptide binding groove that would restrict the binding of poly-acetylated peptides (Fig. S2). Taken together, our modeling of the human PBRM1 BAH domains provide valuable insights into how this protein can recognize histones that will help guide future structural studies.

### Mutation analysis of the PBRM1 BAH domains defines residues required for histone interaction

**Figure 4:**
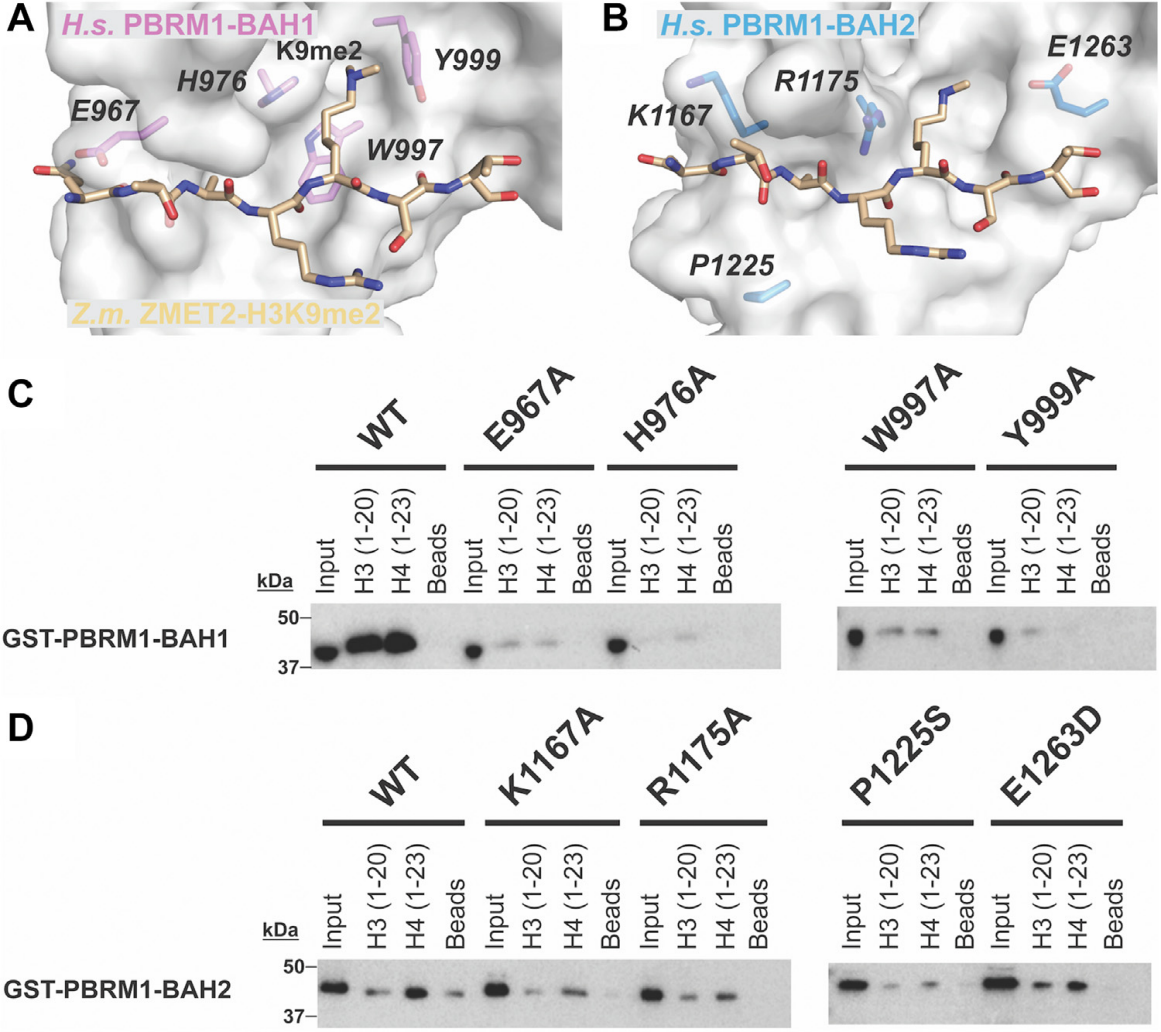
**Mutagenesis of putative histone-binding residues of the BAH1 and BAH2 domains of human PBRM1 reduces histone binding.***A* and *B*, homology models of *H.s.* PBRM1 BAH1 and BAH2 highlighting residues that were mutated (*italics*) due to suspected association with histone tails. *C* and *D*, histone peptide pulldown assays with recombinant GST-PBRM1-BAH1 and BAH2 wild-type (WT) or indicated mutants. Target residues were mutated either to alanine to efficiently disrupt potential histone interactions or mutated to residues found in cancers (BAH2 – P1225S in skin cancer and E1263D in lung cancer). A 1% Input and beads alone (no peptide) were included as controls. Images shown are representative of n ≥ 3 experiments.

Our structural modeling of the PBRM1 BAH domains identified multiple residues within the putative aromatic cage or surface binding groove that were predicted to be important for histone binding (Fig. 4, *A* and *B*). For BAH1, the important residues were H976, W997, and Y999 within the aromatic cage and E967 in the putative binding groove (Fig. 4*A*). Although the BAH2 domain did not appear to contain a canonical aromatic cage, several residues adopted positions similar to those in other BAH histone-binding grooves, for example, K1167 and P1225, and aromatic cages, R1175 and E1263, which suggested that these residues are important for interacting with histones (Fig. 4*B*). Intriguingly, in the COSMIC database, P1225 and E1263 are sites of recurring mutations in skin cancer (P1225S) and lung cancer (E1263D) (Table 1). To determine the importance of these BAH1 and BAH2 residues in histone binding, we mutagenized them to alanine (or to the corresponding cancer-associated mutations for BAH2) in our GST-fusion constructs and repeated our histone peptide-pulldown assays. All four of the BAH1 mutants displayed reduced H3 and H4 peptide binding, with the greatest binding defects observed for the H976A and Y999A mutants within the aromatic cage (Fig. 4*C*). In contrast to the BAH1 mutants, we observed more specific changes in histone binding upon mutating sites in the putative histone binding pocket of the BAH2 domain (Fig. 4*D*). Specifically, there were no broad histone binding defects similar to those observed for the BAH1 mutants; instead, there was an observable reduction in histone binding to the H4 tail for BAH2 proteins harboring K1167A, R1175A, or P1225S mutations (Fig. 4*D*).

**Table 1:** Putative histone binding and/or cancer-associated residues in the human PBRM1 BAH domains

Domain	Position	Putative role	Mutations	Cancer Organ
BAH1	E967	Histone recognition	-	-
**BAH1**	**H976**	**Histone recognition**	**-**	**-**
BAH1	V978	Unknown	V978I	Central Nervous System
BAH1	W997	Histone recognition	-	-
BAH1	Y999	Histone recognition	-	-
BAH1	R1000	Unknown	R1000Q/L/G	Stomach/Skin/Lung
BAH1	P1001	Unknown	P1001Q	Skin
BAH1	N1002	Unknown	N1002D	Thyroid
BAH1	V1017	Unknown	V1017F	Kidney
BAH1	N1024	Histone recognition	-	-
BAH1	V1026	Histone recognition	-	-
BAH1	V1028	Unknown	V1028D	Bile Duct
BAH1	K1030	Histone recognition	-	-
BAH1	A1065	Histone recognition	A1065V	Blood
BAH2	K1167	Histone recognition	-	-
BAH2	R1175	Histone recognition	-	-
BAH2	G1177	Unknown	G1177V	Liver
BAH2	I1195	Histone recognition	-	-
BAH2	I1197	Histone recognition	-	-
BAH2	T1202	Unknown	T1202K	Kidney
BAH2	E1222	Histone recognition	-	-
BAH2	C1224	Histone recognition	-	-
**BAH2**	**P1225**	**Unknown**	**P1225S**	**Skin**
BAH2	M1226	Unknown	M1226V	Large Intestine
BAH2	C1228	Histone recognition	-	-
BAH2	E1263	Histone recognition	E1263D	Lung

Residues targeted for mutagenesis (*i.e.*, H976A and P1225S) in chromatin affinity and quantitative PCR experiments are bolded. The information was derived from the COSMIC database.

Considering the potential for mutations to impair protein folding and/or stability, we next sought to verify that the reduced histone binding observed with our BAH1 and BAH2 mutants was not simply due to drastic changes in protein stability. Thus, we performed differential scanning fluorimetry (DSF) of all of the GST fusions used in this study. These analyses showed that all of the engineered BAH1 and BAH2 mutants had no, or only minimal, changes in melting temperature (Fig. S3).

### Histone-blocking mutations within the PBRM1 BAH domains do not change its overall association on chromatin

**Figure 5:**
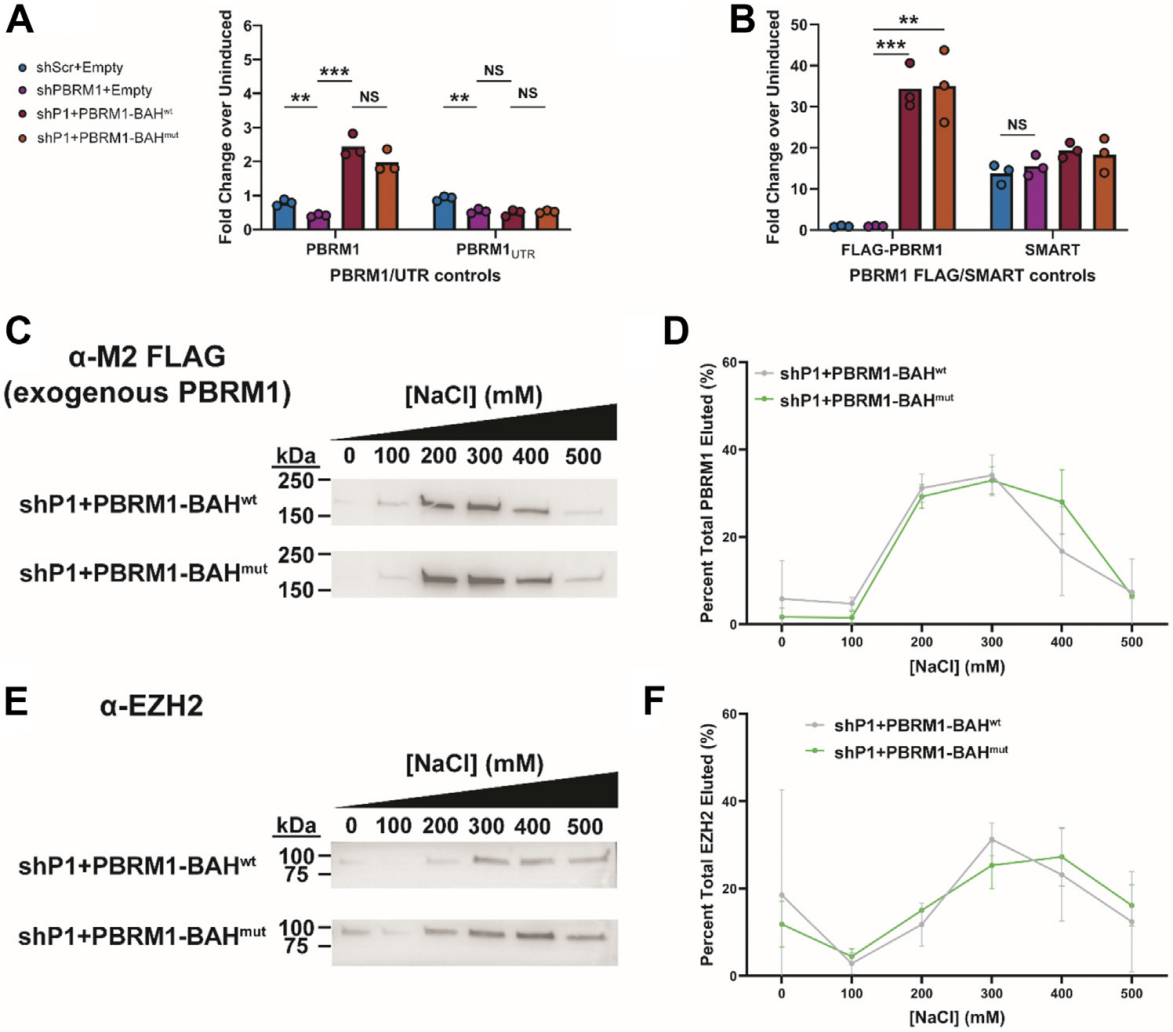
**Mutations that inhibit human PBRM1 BAH1 and BAH2 domain interaction with histones do not significantly impact PBRM1 chromatin association.***A* and *B*, reverse-transcription quantitative PCR (RT-qPCR) of total, endogenous, or exogenous PBRM1 as well as SMART vector control transcripts in HEK293T cells transfected with the following construct(s): (1) scrambled shRNA (shScr), (2) PBRM1 shRNA (shPBRM1), (3) shPBRM1 cotransfected with full-length wild-type PBRM1 (shP1+PBRM1-BAH^wt^), and (4) shPBRM1 cotransfected with PBRM1 containing tandem H976A and P1225S BAH mutations (shP1+PBRM1-BAH^mut^; see legend in 5*A*). *C*–*F*, chromatin affinity assays of HEK293T nuclear extracts from cells transfected with TetOn-inducible shPBRM1 and either PBRM1-BAH^wt^ or PBRM1-BAH^mut^ and induced with doxycycline for 2 days. *C*, Western blots of FLAG-tagged protein (M2 α-FLAG, Sigma) from indicated HEK293T nuclear extracts serially washed with increasing concentrations of NaCl buffer (0–500 mM NaCl in 100 mM increments). *B*, band densitometry of FLAG Western blot signals normalized to total FLAG signal from all wash extracts (Image Studio). *E* and *F*, control chromatin affinity blots and densitometry using the same nuclear extracts and an EZH2 antibody (Cell Signaling, 5246). Images are representative from n ≥ 3 biological replicates.

We next sought to determine whether the histone-binding activity of the PBRM1 BAH domains was important for the ability of PBRM1, and by inference the PBAF complex, to associate with chromatin. To address this question, we first generated a doxycycline-inducible shRNA knockdown of PBMR1 in HEK293T cells. We then co-transfected these cells with a doxycycline-inducible construct containing either full-length 3xFLAG-tagged wild-type PBRM1 (PBRM1-BAH^wt^) or PBRM1 containing mutations in both BAH domains (H976A in BAH1 and P1225S in BAH2) to fully disrupt BAH-histone binding (PBRM1-BAH^mut^) (Fig. S4). The H976A and P1225S mutations were chosen because they had the greatest effect on histone binding along with the least disruption of protein folding as assessed by DSF analysis (see Figs. 4 and S3). Knockdown and complementation of PBRM1 was confirmed by reverse-transcription quantitative PCR (RT-qPCR) with primers specific for either the endogenous or exogenous PBRM1 sequences (Fig. 5, *A* and *B*).

We then tested the effect of the tandem BAH domain mutations on PBRM1 chromatin association using an established salt-based fractionation method for chromatin-binding factors including PBRM1 (38, 42). Slaughter *et. al.* (38) showed that mutations of the PBRM1 BRD2 and BRD4 that prevented binding to acetylated histone also reduced the overall chromatin association of PBRM1. In contrast to those findings, tandem mutation of the BAH domains did not result in any noticeable changes in PBRM1 chromatin association compared to the wild-type protein (Fig. 5, *C* and *D*). As a control, we probed for EZH2 which is a component of the PRC2 complex that writes H3K27me3 (43). EZH2 also did not show a significant change in chromatin association as PBRM1 in the same doxycycline-treated cells (Fig. 5, *E* and *F*). Additionally, we doubled the doxycycline induction time for these cells to exacerbate endogenous PBRM1 depletion, but again the association of exogenous PBRM1 with chromatin appeared to be unaffected by the BAH mutations that prevented histone interaction (Fig. S5, *A* and *B*). Taken together, these data revealed that, although the BAH domains of PBRM1 can bind histones, this binding is not a pre-requisite for PBRM1’s overall ability to associate with chromatin. It is likely that the many other histone- and DNA-binding modules found within PBRM1 and other members of the PBAF complex (44) have more dominant activities in chromatin association and/or targeting.

### Histone-blocking mutations in the BAH domains of PBRM1 result in the dysregulation of a subset of PBRM1 target genes

Although the histone binding activity of the PBRM1 BAH domains was not important for chromatin association, we still asked whether BAH-dependent histone binding was important for the ability of PBRM1/PBAF to regulate transcription. Studies of multiple cell types have defined gene classes and specific genes that are commonly regulated by PBRM1/PBAF (45, 46), thereby providing a set of candidate genes to examine in our aforementioned RT-qPCR-validated doxycycline-inducible PBRM1 knockdown and complementation system. We focused on a subset of target genes that fell into several categories of known PBRM1/PBAF-regulated genes, *i.e.*, genes involved in cell cycle, metabolism, hypoxia, immunity, and adhesion.

**Figure 6:**
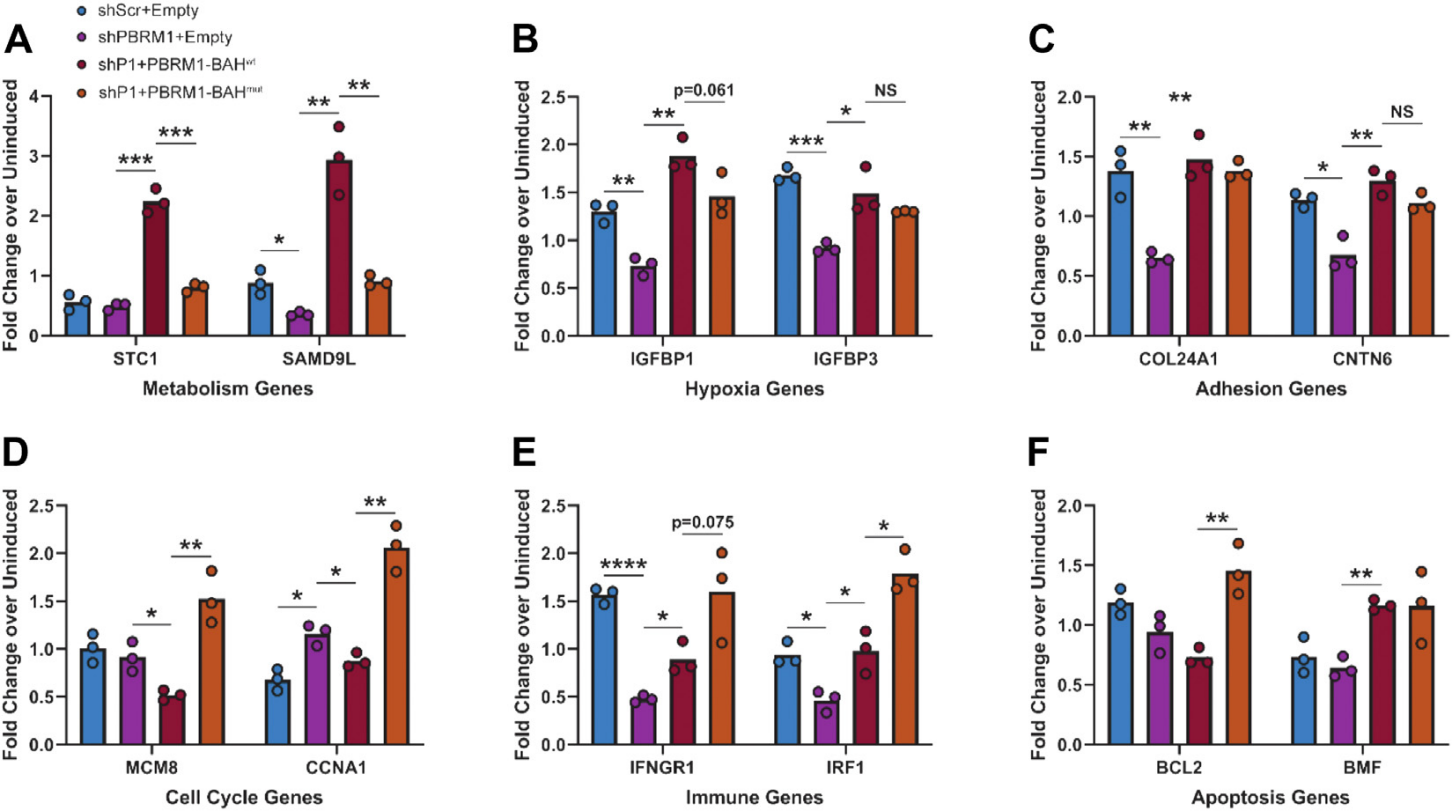
**Mutation of the histone-binding sites of the human PBRM1 BAH1 and BAH2 domains alters transcription of PBRM1-regulated gene families.***A*–*F*, reverse-transcription quantitative PCR (RT-qPCR) of genes linked to PBRM1-dependent regulation in HEK293T cells. Cells were transfected with shRNA and/or FLAG-tagged full-length human wild-type PBRM1 (BAH^wt^) or tandem BAH mutated (BAH^mut^) complementation vectors and induced with doxycycline for 4 days (see legend in 6*A*). The gene classes (*e.g.*, metabolism genes) are listed under the graphs. Average fold-changes are representative from n ≥ 3 biological replicates. Statistical significance from *t* test analyses are reported as *p* ≤ 0.05 (∗), *p* ≤ 0.005 (∗∗), *p* ≤ 0.0005 (∗∗∗), *p* ≤ 0.00005 (∗∗∗∗), and not significant (NS).

As shown in Figure 6*A*, metabolic genes STC1 and SAMD9L were differentially regulated by the absence of endogenous PBRM1. Specially, knockdown of PBRM1 significantly reduced the expression of SAMD9L but not STC1. Exogenous expression of PBRM1-BAH^wt^ in these knockdown cells resulted in a significant increase in the expression of both STC1 and SAMD9L, but intriguingly PBRM1-BAH^mut^ did not upregulate those genes. These results indicated that the BAH domains were important for the proper regulation of the metabolic genes. Similar to expression of SAMD9L, PBRM1-regulated hypoxia genes IGFBP1 and IGFBP3 were downregulated by the absence of PBRM1 (Fig. 6*D*). Both PBRM1-BAH^wt^ and PBRM1-BAH^mut^ were largely capable of rescuing expression of IGFBP3. However, there was an observable (yet nonsignificant) deficiency in IGFBP1 rescue by PBRM1-BAH^mut^. This indicated that, unlike metabolic genes SAMD9L and STC1, regulation of hypoxia genes can be impacted but is less dependent on BAH-histone interactions. Additionally, PBRM1-targeted adhesion genes COL24A1 and CNTN6 were positively regulated upon PBRM1-BAH^wt^ expression, and these genes had slight, nonsignificant deficiencies in PBRM1-BAH^mut^ complementation (Fig. 6*C*). Notably, upregulation of these metabolic, hypoxia, and adhesion associated genes following expression of exogenous PBRM1-BAH^wt^ agreed with findings of Chowdhury *et al.* (45) for Caki-2 clear cell renal carcinoma cells. Our results suggest that the PBRM1 BAH domains are important for promoting expression of PBRM1-linked metabolic genes but not for certain hypoxia or adhesion genes.

While the expression of some PBRM1 targeted genes was not as significantly stimulated by the expression of exogenous PBRM1-BAH^mut^ compared with BAH^wt^, we also observed several genes that were significantly upregulated with the tandem BAH mutant relative to the wild-type control. For example, MCM8 and CCNA1, two cell cycle genes that are normally downregulated in clear cell renal carcinoma Caki-1/2 cells after exogenous PBRM1 expression (45), were significantly upregulated in PBRM1 knockdown cells that expressed PBRM1-BAH^mut^ compared with PBRM1-BAH^wt^ (Fig. 6*D*). Another class of genes we examined was that of immune checkpoint-regulated genes, which include IFNGR1 and IRF1 (both of which are important for T cell recognition of cancer cells) (46). As shown in Figure 6*E*, IFNGR1 and IRF1 were both downregulated by PBRM1 depletion and largely rescued by expression of PBRM1-BAH^wt^. However, similar to expression of MCM8 and CCNA1, complementation with PBRM1-BAH^mut^ led to a further increase in the expression of IFNGR1 and IRF1 (Fig. 6*E*). Finally, one gene class showed conflicting results associated with expression of PBRM1-BAH^mut^. This class included apoptotic genes BCL2 and BMF, which are normally upregulated by PBRM1 in ccRCC cells (45). In contrast, loss of PBRM1 did not have an effect on BCL2 and BMF expression in HEK293T cells (Fig. 6*F*). Similar to the aforesaid cell cycle and immune checkpoint genes (Fig. 6*F*), BCL2 expression was significantly upregulated in the knockdown line that expressed the tandem BAH mutant form of PBRM1 (Fig. 6*F*). These results indicated that the PBRM1 BAH domains were indeed important to the expression of these five genes but in a manner that limits their expression. Conversely, BMF was unaffected by PBRM1 knockdown in HEK293T cells and upregulated equivalently in both the PBRM1-BAH^wt^ and PBRM1-BAH^mut^ cell lines (Fig. 6*F*). We surmised that BCL2 and BMF are likely controlled differently in HEK293T cells, which could explain why these two genes were not as responsive to PBRM1 loss or gain as observed for other cell types (45).

Altogether, the foregoing transcriptional analyses provided important evidence that BAH-histone recognition is indeed required for proper PBRM1-dependent transcriptional regulation of a subset of genes that are closely associated with renal cancer progression, particularly metabolism genes such as STC1 and SAMD9L.

## Discussion

PBRM1 is a large, multi-domain protein that has been widely linked to the onset of clear cell renal cell carcinomas (26). PBRM1 contains multiple putative histone reader domains including two relatively uncharacterized BAH domains. In this study, we found that the PBRM1 BAH domains were capable of binding to histones and recognize mainly unmodified H3 and H4 tails. The BAH2 was competent in histone binding but may serve as an auxiliary histone binding unit to the BAH1 or have different histone binding preferences in cells. Poly-acetylation of the H3 and H4 tails appeared to substantially perturb the association of the BAH domains, possibly due to the disruption of electrostatic interactions between the protein and histone tails. Intriguingly, BAH binding to histones was perturbed by H3K27me3, a modification critical for the recruitment of polycomb repressive complexes and gene silencing. Methylation of H3K27 could create steric hindrance or prevent the formation of hydrogen bonding between the unmodified lysine and residues lining the PBRM1 BAH binding pockets, akin to how methylation of H3K27 disrupts recognition by AF10’s hydrogen-bond acceptor lined PZP pocket (47). This finding is consistent with the notion that PBRM1/PBAF is generally a positive regulator of transcription. Thus, H3K27me3 may function as a gatekeeper to prevent the PBAF complex from activating repressed genes. Loss of this positive gene regulation, either by suppression or mutation of PBRM1, appears to support the expression of genes that are critical for cancer functions including hypoxia-dependent growth and survival, bypass of cell-cycle checkpoints, and aberrant adhesion that promotes tumor expansion (45, 46, 48).

As a family, the BAH domains are less well characterized compared with other histone readers like the PHD and BRD families. Our computational modeling of the human PBRM1 BAH domains is one of the first examples to show how these reader domains likely recognize histones. We suspect that there is a conserved mode of histone binding among BAH domains (Fig. 2) that involves the tail lying in the broad front-facing groove along the BAH surfaces and a critical histone lysine binding in the putative aromatic or noncanonical pockets. This binding mode would be similar to histone binding to PHDs (49) and BRDs (50) due to the involvement of an aromatic pocket, but the overall BAH folding and groove appear to be unique and may enable different histone binding specificities. Additionally, there may even be different histone binding specificities between the two PBRM1 BAH domains due to variations in the aromatic cage region (Fig. 3). Specifically, BAH2 has an R1175, I1195, and E1263 where aromatic residues are often found such as H976, W997, and Y999 in BAH1. The presence of charged residues in the BAH2 pocket may explain why it favors binding of unmodified as opposed to acetylated histone tails (Fig. 1, *B*–*D*). However, we have not yet identified a preferred histone modification binding state(s) for the PBRM1 BAH1 domain. Future efforts will be focused on developing high-throughput *in vitro* screening technology for nucleosomal binding which may yield clearer insights into how the PBRM1 BAH domains specifically bind to histone tails and/or their modifications.

From our initial histone binding screens and structural homology modeling, we identified several residues in the PBRM1 BAH domains that are likely involved in histone binding, including some residues that are mutated in cancer (Fig. 4). For instance, mutation of P1225S in the PBRM1 BAH2 domain severely ablated histone binding (Fig. 4*D*). P1225 is positioned on the surface of the putative histone binding groove of the BAH2 domain (Fig. 4*B*) and mutation of this residue to serine likely perturbs the overall shape and polarity of the pocket, thereby disrupting histone binding. Thus, the P1225S mutation in certain skin cancers (COSMIC) could promote oncogenic development by disrupting PBRM1/PBAF-dependent histone binding, a conjecture that requires investigation. Additionally, we followed the effects of H976A and P1225S mutations in PBRM1 expressed in cells. Although perturbing BAH-histone interaction did not result in defects in overall PBRM1 chromatin binding (Fig. 5), the mutations did appear to significantly affect target gene regulation. We speculate that either the PBRM1 BRDs have a more dominant function in overall PBRM1/PBAF chromatin recruitment (38, 42) or that other domains in PBRM1, such as the putative C-terminal DNA-binding HMG domain, have yet to be clarified major functions in chromatin recruitment. Additionally, the other subunits of the BAF complex containing histone and DNA-binding domains could support PBRM1’s retention on chromatin in the absence of PBRM1 BAH-mediated histone binding (26).

The RT-qPCR results of our BAH mutants revealed multiple gene classes (with links to PBRM1-associated cancers) that became dysregulated upon BAH1 and BAH2 mutation (Fig. 6). Importantly, genes involved in metabolic and hypoxia pathways that are critical for controlling oncogenic development were no longer induced by PBRM1 that contained tandem BAH histone-binding mutations (Fig. 6, *A* and *B*). Conversely, expression of PBRM1 containing tandem BAH histone-binding mutations resulted in even greater activation of certain cell cycle and immune responsive genes (Fig. 6, *D* and *E*). Our RT-qPCR data altogether paint PBRM1 as a multifactorial gene regulator that may have roles distinguished by different functional domains, such as the BAH domains that drive metabolic and hypoxia-dependent responses whereas other pathways may be driven by the bromo and HMG domains. Moreover, PBAF function is also dependent on other subunits and more globally by transcriptional and epigenetic activators or repressors, especially those that respond to changes in cellular homeostasis or external signaling (26, 44). Thus, the BAH domains of PBRM1 may contribute to fine-tuning gene regulation after PBAF is recruited to target genes in a context-dependent manner.

In yeast, Schlichter *et. al* (51). showed that PBRM1’s homolog RSC1 is important for the nucleosome remodeling function of the RSC complex. That earlier study and ours, therefore, document an evolutionarily conserved function of the BAH domains in chromatin regulation that is likely mediated by histone interaction. Although our evidence points to histone binding by the BAH1 and BAH2, we do not rule out other suggested targets of these domains (52).

Yuan *et. al.* (53) recently obtained cryo-EM structures of the human PBAF complex that point to PBRM1 being oriented towards histone tails. In these structures, only a short portion of the C-terminus of PBRM1 could be resolved, and it lacked density for the BRDs, HMG, and BAHs of PBRM1 (53). Combining the cryo-EM structural information with our results showing direct histone tail interaction by not only the BRD but also the BAH domains of PBRM1, we can envision that PBRM1 facilitates chromatin unwinding by binding to and guiding the BAF complex to specific gene start sites *via* its histone-reading modules. We attempted to determine the impact of our histone-blocking BAH mutations on PBRM1’s chromatin localization and occupancy by ChIP. However, we faced significant technical issues that were likely due to the masking of the FLAG epitope by formaldehyde cross-links and the high mobility of PBAF complexes (data not shown). Further genomic-level probing of PBRM1 histone-reading mutants, potentially using CUT&RUN or alternative PBAF targets such as PHF10 (54), will be required to clearly demonstrate how this protein and its different reader domains contribute to PBAF localization. Nonetheless, our results show that the PBRM1 BAH1 and BAH2 are competent in binding histone tails which is likely by a conserved BAH family fold. Mutations of the histone-reading residues of these BAH domains cause defects in the expression of important PBRM1-targeted genes. Further investigation of how mutations in the histone-binding regions of the PBRM1 BAH1 and BAH2 domains disrupt transcriptional networks associated with cancer development, particularly clear cell renal carcinoma, should drive the design of novel treatments for PBRM1-linked cancers.

## Experimental procedures

### Protein modeling

Experimentally determined BAH structures suitable as templates for modeling of PBRM1 BAH domains were identified using HHpred. Due to low sequence similarity, HHpred secondary structure predictions and analysis of the experimentally-determined BAH domain structures were used to identify a “core” fold conserved among these BAH domains and to evaluate template suitability. We selected PDB 1W4S, the BAH domain of *Gallus gallus* PBRM1, as the main template for the human BAH domains from *Homo sapiens* PBRM1. We also utilized PDB 4FT4, the BAH domain from *Z. mays* ZMET2, for modeling the histone peptide binding. In the threadings generated by HHpred, the template 1W4S BAH domain from *G.g.* PBRM1 shared only 36.2% sequence identity with the modeled *H.s.* BAH2 domain, whereas it shared 98.6% sequence identity with the modeled *H.s.* BAH1 domain. These threadings were used with HHpred to develop structural models for the *H.s.* PBRM1 BAH domains (40).

### Protein expression and purification

GST-tagged PBRM1 BAH1 (residues 932–1108) and BAH2 (residues 1136–1293) proteins were expressed from the pGEX-6P1 plasmid in SoluBL21 bacterial cells and purified, with some modification (39). Briefly, bacteria were grown to an OD_600_ of ∼0.3, then shifted to 16 °C and induced overnight with 0.2 mM IPTG. Post induction, the cells were harvested, lysed, and sonicated as described (39). The lysis buffer was modified for BAH2 constructs to include 10% hexanediol to improve yield. After dialysis, proteins were quantified by Bradford assay. Site-directed mutagenesis was performed with the QuikChange II kit (Agilent, 200523) and mutations were confirmed by sequencing. Purifications of mutant proteins were performed following the same strategy as used for the wild type proteins.

### In solution peptide pulldowns

Peptide pulldown assays were performed as previously described, with slight modification (39). Standard buffer conditions were used (50 mM Tris pH 8.0, 300 mM NaCl, 0.1% NP-40, 0.5% BSA) except for the BAH1 constructs, for which 10% hexanediol was added to the assay to reduce non-specific aggregation and bead binding. Briefly, 0.1 μM of GST-PBRM1 proteins were incubated at 4 °C with indicated biotinylated peptides at 1 μM. The peptides were then captured using Pierce Streptavidin Magnetic Beads (Thermo). After successive washes in peptide binding buffer, the peptide-protein complexes were denatured in SDS-loading dye and subjected to gel electrophoresis and western blotting for detection of the GST-tag on the PBRM1 proteins (in-house antibody; 1:5000).

### Tissue culture

HEK293T cells were used in this study with PBRM1 knockdown lines generated by transfection with Lipofectamine 2000 according to the manufacturer’s protocol (ThermoFisher, 11668030). SMARTinducible shRNA with a non-targeting scramble sequence (Horizon, VSC11653) or shRNA against PBRM1 (Horizon, V3SH11252- 228978129) were used to generate knockdown lines. Knockdown was evaluated by standard Western blot using anti-PBRM1 antibody (Millipore, ABE70) with Lamin B1 as a control (Abcam, ab16048). We selected 2 days of doxycyline treatment (1 μg/ml) for chromatin association assays due to optimal detection of exogenous PBRM1 and 4 days of doxycycline treatment (1 μg/ml, replaced supplemented media at 2 days) for RT-qPCR experiments due to optimal knockdown. To generate the PBRM1 rescue lines, TetOn plasmids compatible with the SMART plasmids carrying empty, wild type, or BAH1/2 mutant FLAG-tagged PBRM1 (H976A + P1225S) were transfected as before with Lipofectamine 2000 into the knockdown and scrambled cell lines. After doxycycline co-induction of the PBRM1 and shRNA plasmids for 2 to 4 days, expression of the FLAG-tagged constructs was assessed by standard Western blot using anti-FLAG M2 antibody (Sigma, F1804). Since the PBRM1 antibody could not distinguish endogenous from exogenous PBRM1, RT-qPCR was used to determine knockdown efficiency in the rescue lines.

### RNA expression analysis

RNA expression analysis was performed by isolating RNA with TRIZOL according to the manufacturer’s protocol (ThermoFisher, 15596026) followed by DNase treatment and repurification. The purified RNA was reverse transcribed using the SuperScript III kit according to manufacturer’s protocol (ThermoFisher, 18080044) and then used in RT-qPCR reactions with gene specific primers and iTaq SYBR Green mix (BioRad, 1725120). Reactions and analyses were performed in an ABI QuantStudio seven instrument and software. We determined the fold change by first normalizing variable targets against GAPDH and then finding the difference of these values against uninduced samples using the MIQE guidelines. See Table S1 for primers.

### Protein stability analysis

Protein stability was assessed by nano differential scanning fluorimetry (Nanotemper DSF) according to the manufacturer’s protocol. Thermal denaturation was performed and measured in 1 °C/min steps from 20 to 95 °C. Stability was determined by measuring tryptophan and tyrosine fluorescence signal during the temperature rise and normalized as a ratio between 330 nm and 350 nm signal to control for baseline wavelength scattering. The temperature of inflection, defined at the point with ∼50% loss of signal from baseline, is reported.

### Data and statistical analysis

All blots shown are representative images of at least three experiments, and all quantifications (nanoDSF and RT-qPCR measurements) were derived from at least three experiments, including two biological replicates for cell culture experiments, with the average and standard deviation shown.

## Data availability

The authors confirm that the data supporting the findings of this study are available within the article and its supplementary information. Raw data files are available upon request and original Western blot images used in this study are presented in Figs. S6–S8.

## Supporting information

This article contains supporting information.

## Conflict of interest

B. D. S. is a co-founder and BOD member of EpiCypher, Inc.

## References

[1] Ramakrishnan V. (1997). Histone structure and the organization of the nucleosome. Annu. Rev. Biophys. Biomol. Struct..

[2] Luger K., Mäder A.W., Richmond R.K., Sargent D.F., Richmond T.J. (1997). Crystal structure of the nucleosome core particle at 2.8 Å resolution. Nature.

[3] Strahl B.D., Allis C.D. (2000). The language of covalent histone modifications. Nature.

[4] Rando O.J., Chang H.Y. (2009). Genome-wide views of chromatin structure. Annu. Rev. Biochem..

[5] Su Z., Denu J.M. (2016). Reading the combinatorial histone language. ACS Chem. Biol..

[6] Rothbart S.B., Strahl B.D. (2014). Interpreting the language of histone and DNA modifications. Biochim. Biophys. Acta.

[7] Suganuma T., Workman J.L. (2011). Signals and combinatorial functions of histone modifications. Annu. Rev. Biochem..

[8] Patel D.J., Wang Z. (2013). Readout of epigenetic modifications. Annu. Rev. Biochem..

[9] Sharma R., Zhou M.-M. (2015). Partners in crime: the role of tandem modules in gene transcription: partners in crime. Protein Sci..

[10] Mansfield R.E., Musselman C.A., Kwan A.H., Oliver S.S., Garske A.L., Davrazou F. (2011). Plant Homeodomain (PHD) Fingers of CHD4 are histone H3-binding modules with preference for unmodified H3K4 and methylated H3K9. J. Biol. Chem..

[11] Huether R., Dong L., Chen X., Wu G., Parker M., Wei L. (2014). The landscape of somatic mutations in epigenetic regulators across 1,000 paediatric cancer genomes. Nat. Commun..

[12] Dawson M.A. (2017). The cancer epigenome: concepts, challenges, and therapeutic opportunities. Science.

[13] Andrews F.H., Strahl B.D., Kutateladze T.G. (2016). Insights into newly discovered marks and readers of epigenetic information. Nat. Chem. Biol..

[14] Finn R.D., Attwood T.K., Babbitt P.C., Bateman A., Bork P., Bridge A.J. (2017). InterPro in 2017-beyond protein family and domain annotations. Nucleic Acids Res..

[15] Yang N., Xu R.-M. (2013). Structure and function of the BAH domain in chromatin biology. Crit. Rev. Biochem. Mol. Biol..

[16] Armache K.-J., Garlick J.D., Canzio D., Narlikar G.J., Kingston R.E. (2011). Structural basis of silencing: Sir3 BAH domain in complex with a nucleosome at 3.0 Å resolution. Science.

[17] Kuo A.J., Song J., Cheung P., Ishibe-Murakami S., Yamazoe S., Chen J.K. (2012). The BAH domain of ORC1 links H4K20me2 to DNA replication licensing and Meier–Gorlin syndrome. Nature.

[18] Hou Z., Danzer J.R., Fox C.A., Keck J.L. (2006). Structure of the Sir3 protein bromo adjacent homology (BAH) domain from S. cerevisiae at 1.95 A resolution. Protein Sci..

[19] Chambers A.L., Pearl L.H., Oliver A.W., Downs J.A. (2013). The BAH domain of Rsc2 is a histone H3 binding domain. Nucleic Acids Res..

[20] Du J., Zhong X., Bernatavichute Y.V., Stroud H., Feng S., Caro E. (2012). Dual binding of chromomethylase domains to H3K9me2-containing nucleosomes directs DNA methylation in plants. Cell.

[21] Zhao D., Zhang X., Guan H., Xiong X., Shi X., Deng H. (2016). The BAH domain of BAHD1 is a histone H3K27me3 reader. Protein Cell.

[22] Thompson M. (2009). Polybromo-1: the chromatin targeting subunit of the PBAF complex. Biochimie.

[23] Pontén F., Jirström K., Uhlen M. (2008). The human protein Atlas--a tool for pathology. J. Pathol..

[24] Uhlén M., Fagerberg L., Hallström B.M., Lindskog C., Oksvold P., Mardinoglu A. (2015). Proteomics. Tissue-based map of the human proteome. Science.

[25] Charlop-Powers Z., Zeng L., Zhang Q., Zhou M.-M. (2010). Structural insights into selective histone H3 recognition by the human Polybromo bromodomain 2. Cell Res..

[26] Hodges C., Kirkland J.G., Crabtree G.R. (2016). The many roles of BAF (mSWI/SNF) and PBAF complexes in cancer. Cold Spring Harb. Perspect. Med..

[27] Helming K.C., Wang X., Roberts C.W.M. (2014). Vulnerabilities of mutant SWI/SNF complexes in cancer. Cancer Cell.

[28] Shain A.H., Pollack J.R. (2013). The spectrum of SWI/SNF mutations, ubiquitous in human cancers. PLoS One.

[29] Varela I., Tarpey P., Raine K., Huang D., Ong C.K., Stephens P. (2011). Exome sequencing identifies frequent mutation of the SWI/SNF complex gene PBRM1 in renal carcinoma. Nature.

[30] Hopson S., Thompson M.J. (2017). BAF180: its roles in DNA repair and consequences in cancer. ACS Chem. Biol..

[31] Goodwin G.H., Nicolas R.H. (2001). The BAH domain, polybromo and the RSC chromatin remodelling complex. Gene.

[32] Cairns B.R., Schlichter A., Erdjument-Bromage H., Tempst P., Kornberg R.D., Winston F. (1999). Two functionally distinct forms of the RSC nucleosome-remodeling complex, containing essential AT hook, BAH, and bromodomains. Mol. Cell.

[33] Oliver A.W., Jones S.A., Roe S.M., Matthews S., Goodwin G.H., Pearl L.H. (2005). Crystal structure of the proximal BAH domain of the polybromo protein. Biochem. J..

[34] Chambers A.L., Brownlee P.M., Durley S.C., Beacham T., Kent N.A., Downs J.A. (2012). The two different isoforms of the RSC chromatin remodeling complex play distinct roles in DNA damage responses. PLoS One.

[35] Kent N.A., Chambers A.L., Downs J.A. (2007). Dual chromatin remodeling roles for RSC during DNA double strand break induction and repair at the yeast MAT locus. J. Biol. Chem..

[36] Shim E.Y., Hong S.J., Oum J.-H., Yanez Y., Zhang Y., Lee S.E. (2007). RSC mobilizes nucleosomes to improve accessibility of repair machinery to the damaged chromatin. Mol. Cell. Biol..

[37] Liao L., Alicea-Velázquez N.L., Langbein L., Niu X., Cai W., Cho E.-A. (2019). High affinity binding of H3K14ac through collaboration of bromodomains 2, 4 and 5 is critical for the molecular and tumor suppressor functions of PBRM1. Mol. Oncol..

[38] Slaughter M.J., Shanle E.K., McFadden A.W., Hollis E.S., Suttle L.E., Strahl B.D. (2018). PBRM1 bromodomains variably influence nucleosome interactions and cellular function. J. Biol. Chem..

[39] Petell C.J., Pham A.T., Skela J., Strahl B.D. (2019). Improved methods for the detection of histone interactions with peptide microarrays. Sci. Rep..

[40] Söding J., Biegert A., Lupas A.N. (2005). The HHpred interactive server for protein homology detection and structure prediction. Nucleic Acids Res..

[41] McDaniel S.L., Fligor J.E., Ruan C., Cui H., Bridgers J.B., DiFiore J.V. (2016). Combinatorial histone readout by the dual plant homeodomain (PHD) Fingers of Rco1 Mediates Rpd3S chromatin recruitment and the maintenance of transcriptional fidelity. J. Biol. Chem..

[42] Porter E.G., Dykhuizen E.C. (2017). Individual bromodomains of polybromo-1 contribute to chromatin association and tumor suppression in clear cell renal carcinoma. J. Biol. Chem..

[43] Margueron R., Justin N., Ohno K., Sharpe M.L., Son J., Drury W.J. (2009). Role of the polycomb protein EED in the propagation of repressive histone marks. Nature.

[44] Ho P.J., Lloyd S.M., Bao X. (2019). Unwinding chromatin at the right places: how BAF is targeted to specific genomic locations during development. Development.

[45] Chowdhury B., Porter E.G., Stewart J.C., Ferreira C.R., Schipma M.J., Dykhuizen E.C. (2016). PBRM1 regulates the expression of genes involved in metabolism and cell adhesion in renal clear cell carcinoma. PLoS One.

[46] Liu X.-D., Kong W., Peterson C.B., McGrail D.J., Hoang A., Zhang X. (2020). PBRM1 loss defines a nonimmunogenic tumor phenotype associated with checkpoint inhibitor resistance in renal carcinoma. Nat. Commun..

[47] Chen S., Yang Z., Wilkinson A.W., Deshpande A.J., Sidoli S., Krajewski K. (2015). The PZP domain of AF10 Senses unmodified H3K27 to regulate DOT1L-mediated methylation of H3K79. Mol. Cell.

[48] Yang J., Nie J., Ma X., Wei Y., Peng Y., Wei X. (2019). Targeting PI3K in cancer: mechanisms and advances in clinical trials. Mol. Cancer.

[49] Sanchez R., Zhou M.-M. (2011). The PHD finger: a versatile epigenome reader. Trends Biochem. Sci..

[50] Filippakopoulos P., Picaud S., Mangos M., Keates T., Lambert J.-P., Barsyte-Lovejoy D. (2012). Histone recognition and large-scale structural analysis of the human bromodomain family. Cell.

[51] Schlichter A., Kasten M.M., Parnell T.J., Cairns B.R. (2020). Specialization of the chromatin remodeler RSC to mobilize partially-unwrapped nucleosomes. Elife.

[52] Karki M., Jangid R.K., Anish R., Seervai R.N.H., Bertocchio J.-P., Hotta T. (2021). A cytoskeletal function for PBRM1 reading methylated microtubules. Sci. Adv..

[53] Yuan J., Chen K., Zhang W., Chen Z. (2022). Structure of human chromatin-remodelling PBAF complex bound to a nucleosome. Nature.

[54] De Silva S.M., Dhiman A., Sood S., Mercedes K.F., Simmons W.J., Henen M.A. (2023). PBRM1 bromodomains associate with RNA to facilitate chromatin association. Nucleic Acids Res..

