# Supplementary material for: The bromo-adjacent homology domains of PBRM1 associate with histone tails and contribute to PBAF-mediated gene regulation
Source: J Biol Chem. 2023 Jun 30;299(8):104996. doi: 10.1016/j.jbc.2023.104996 (PMC10425938; doi:10.1016/j.jbc.2023.104996)
Supplement: Supporting Figures S1–S8 [file mmc2.pdf]

Supporting Information

The bromo-adjacent homology (BAH) domains of PBRM1 associate with histone tails and contribute to PBAF-mediated gene regulation

Christopher J. Petell[#1,2], Nathaniel T. Burkholder[#1,2], Paloma A. Ruiz[1], Jessica Skela[1], Jake R. Foreman[1], Lauren E. Southwell[1], Brenda R. Temple[2,3], Krzysztof Krajewski[1,2], and Brian D. Strahl[1,2]*

[1]Department of Biochemistry and Biophysics, 120 Mason Farm Rd, University of North Carolina at Chapel Hill, NC, USA 27599; USA

[2]UNC Lineberger Comprehensive Cancer Center, 450 West Drive, University of North Carolina at Chapel Hill, NC, USA 27599; USA

[3]R L Juliano Structural Bioinformatics Core Facility, 120 Mason Farm Rd, University of North Carolina at Chapel Hill, NC, USA 27599; USA

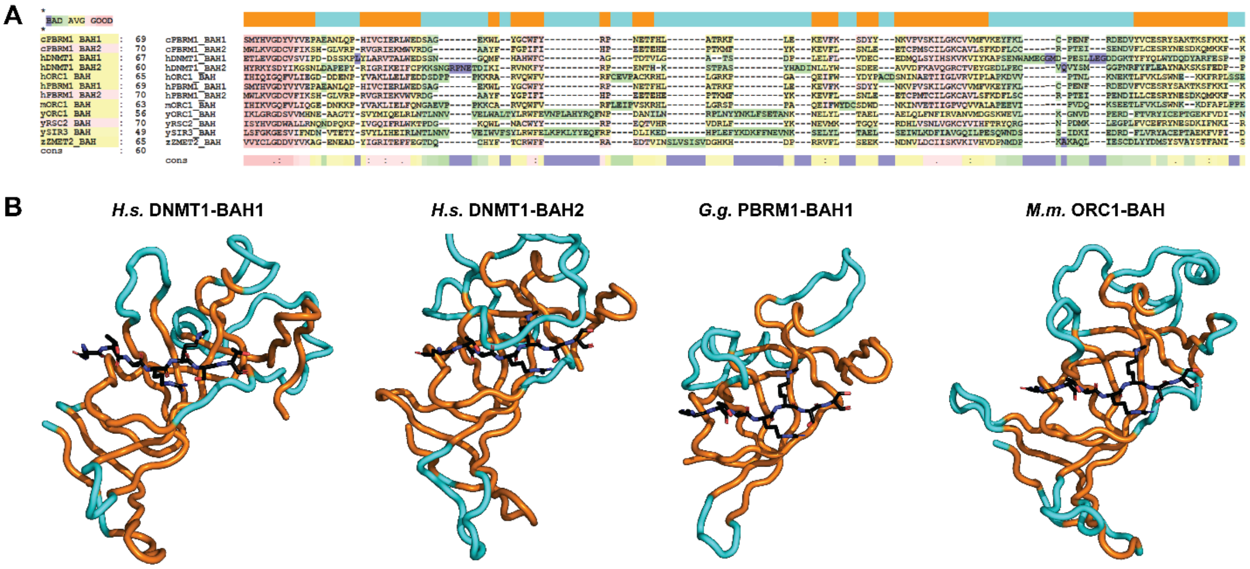

Figure S1. Sequence and structural similarity of BAH domains highlighting a structurally conserved "core". *A,* T-COFFEE amino acid sequence alignment of BAH domains of several different proteins from various species. Structurally conserved "core" (orange) and poorly conserved variable loops (teal) are highlighted. *B,* Structural alignment and analysis of the BAH domains from *Homo sapiens* DNMT1 (PDB:3SWR), *Gallus gallus* PBRM1 (PDB:1W4S), and *Mus musculus* ORC1 (PDB:4DOV) revealed a conserved "core" and variable loop regions. H3K9me2-bound peptide (black) from *Zea mays* ZMET2 structure was modeled within conserved putative BAH binding pockets.

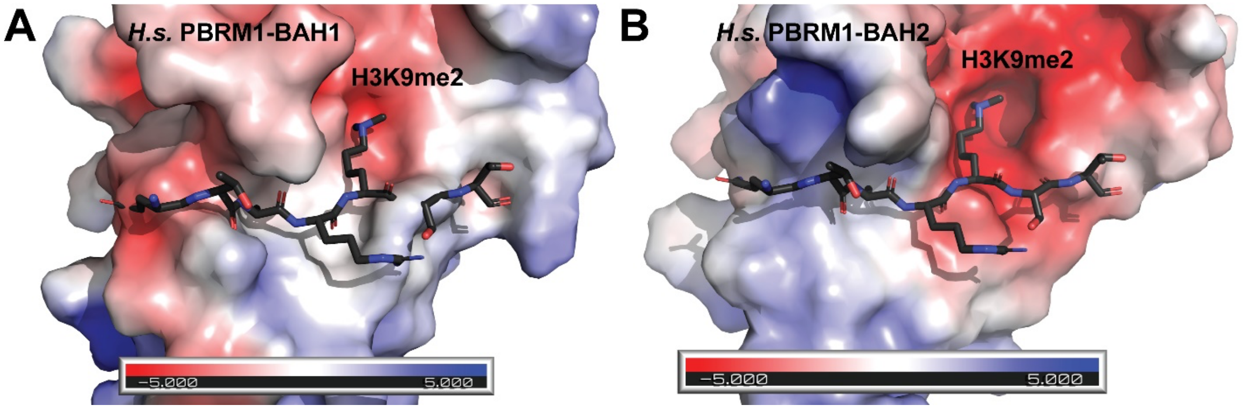

Figure S2. Electrostatic maps of human PBRM1-BAH models highlighting putative histone binding pocket. *A-B,* HHPRED-generated structural homology models of the *Homo sapiens* PBRM1-BAH1/2 domains. Surfaces predicted to be positively (blue) and negatively (red) charged are displayed (Pymol). H3K9me2-bound peptide (black) from *Zea mays* ZMET2 structure was modeled within conserved putative PBRM1-BAH1/2 binding pockets.

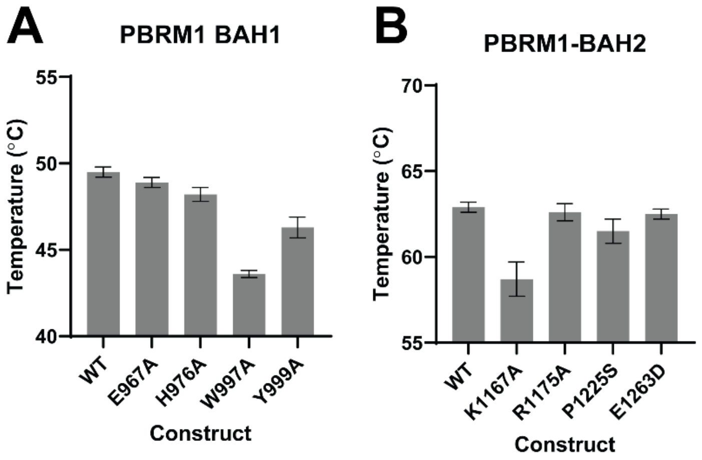

Figure S3. Thermostability of purified PBRM1-BAH1/2 proteins. *A-B,* Melting temperatures of indicated PBRM1 BAH1 or BAH2 proteins derived from nano differential scanning fluorimetry. Averages and standard deviations represent technical replicates of $n \geq 3$.

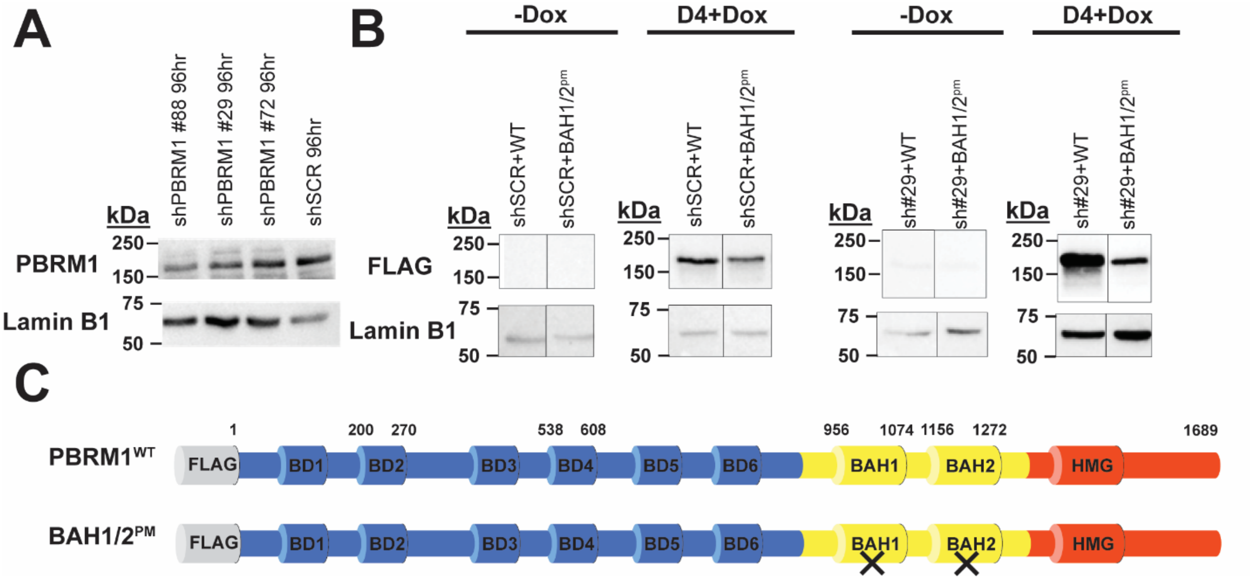

Figure S4. Expression of endogenous and rescue PBRM1 in shPBRM1 cell lines. *A-B,* Western blotting of PBRM1 in inducible shPBRM1 HEK293T cells. *A,* Expression of endogenous PBRM1 (Millipore, ABE70) and Lamin B1 (abcam, ab16048) in cells transduced with indicated shRNAs (#88, #29, #72, scrambled or SCR) and induced for four days with doxycycline. Subsequent rescue experiments used the shPBRM1 #29-transduced cell line as a base (Fig. 5/6). *B,* Expression of FLAG-tagged PBRM1 and Lamin B1 in shSCR and shPBRM1 #29 cells transduced with indicated inducible PBRM1 rescue vectors after four days of doxycycline induction. Shown are spliced images (delineated by black borders between lanes) taken from western blots probed for FLAG or Lamin B1 in the shSCR and shPBRM1 #29 samples. Westerns for FLAG or Lamin B1 all shared the same exposure times. *C,* Domain diagrams of FLAG-tagged PBRM1 proteins for rescue experiments outlined in Fig. S4B. Indicated pocket mutations (H976A and P1225S) are illustrated as X's.

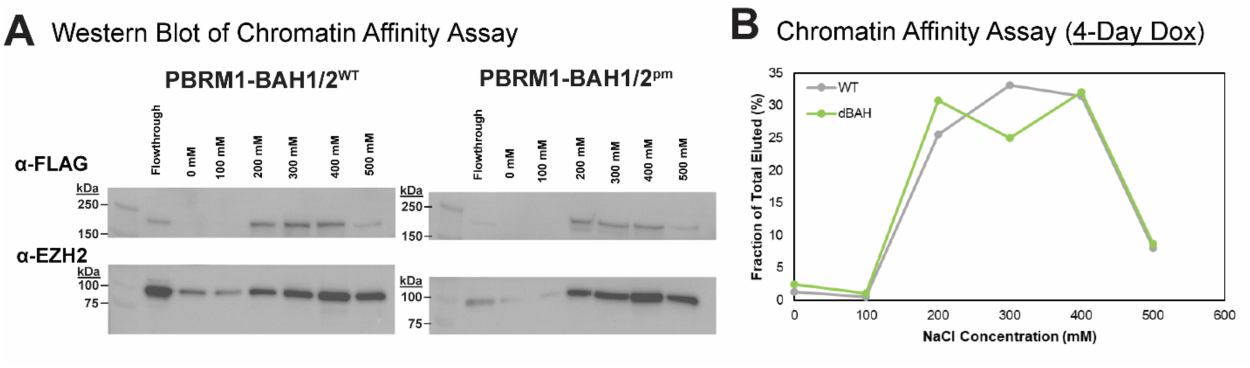

Figure S5. Chromatin affinity assay of 3xFLAG-PBRM1 after four days of doxycycline induction. *A-B,* NaCl fractionation of chromatin from 4-day doxycycline inductions (1 µg/mL; doxycycline media changed after two days) of HEK293T cells co-expressing sh#29-PBRM1 shRNA and 3xFLAG-PBRM1-BAH^{wt} vs. 3xFLAG-PBRM1-BAH^{mut}. Western blots of FLAG (M2 antibody) were performed and analyzed by densitometry for determining percent fraction eluted.

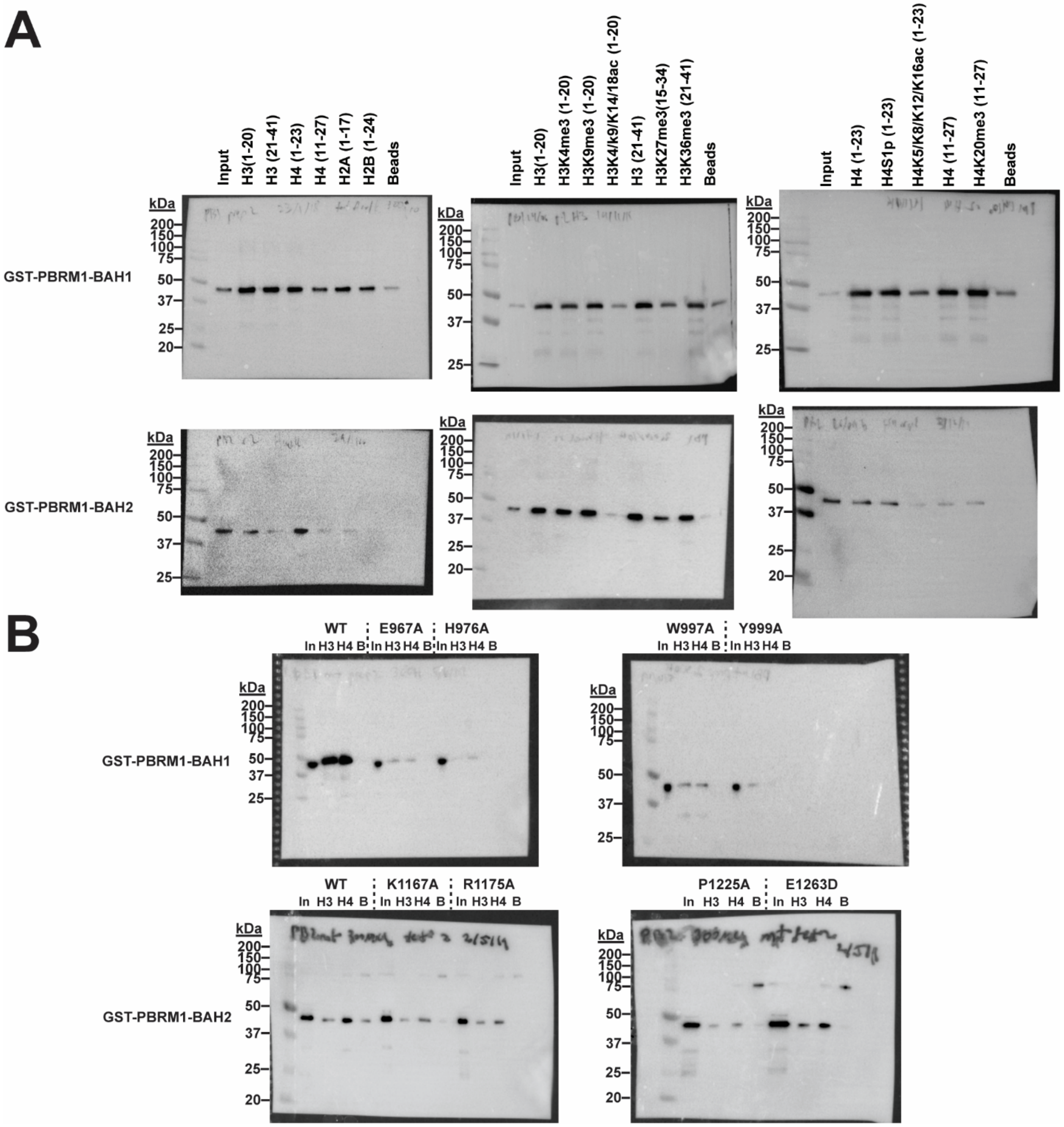

Figure S6. Full western blots of peptide pulldown assays using purified GST-PBRM1-BAH1/2 proteins. *A,* Full blots for images in Figure 1 using wild-type GST-PBRM1-BAH proteins. *B,* Full blots for images in Figure 4 using mutant GST-PBRM1-BAH proteins. Lanes are labeled as In (input), H3 (H3 1-20), H4 (H4 1-23) and B (beads only control).

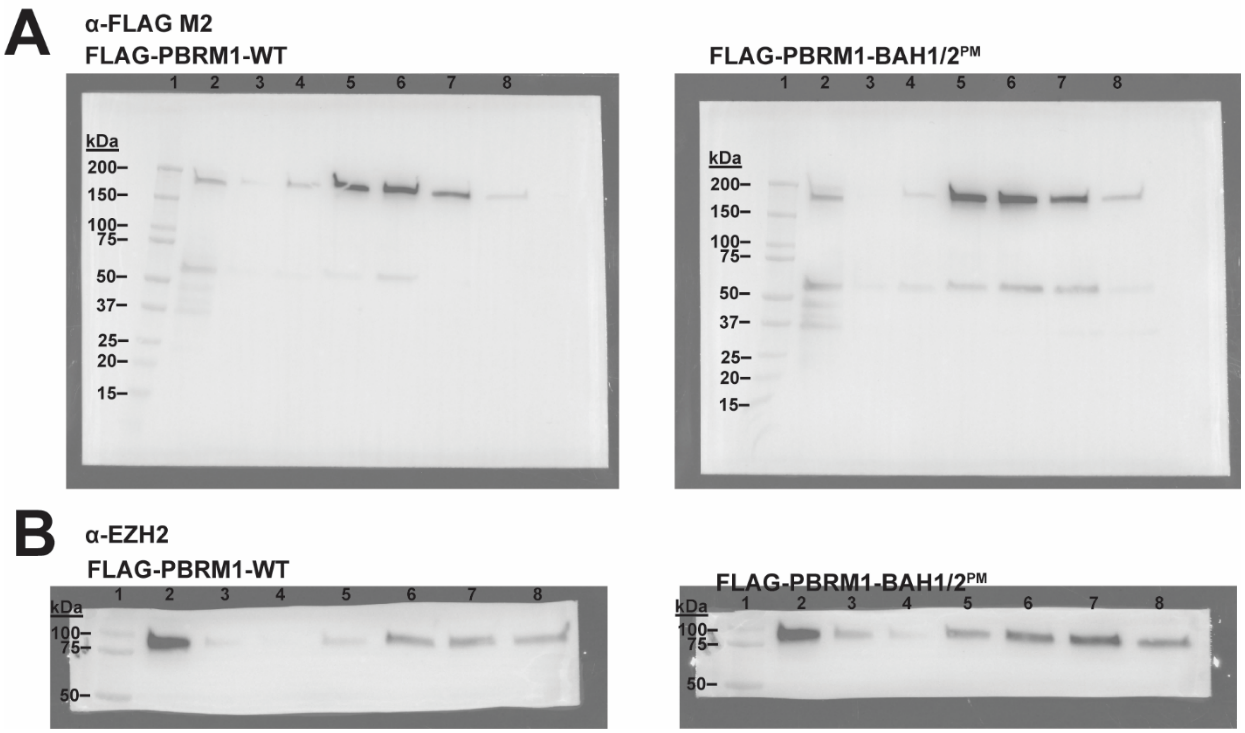

Figure S7. Chromatin affinity assay western blots of 3xFLAG-PBRM1 from HEK293T cells. *A-B,* Full western blot images of chromatin salt fractionations from 2-day doxycycline-induced (1 μg/mL) HEK293T cells co-expressing shPBRM1 #29 and 3xFLAG-PBRM1 WT, BAH, and BRD mutants used to generate panels in Figure 5. *A,* Western blots using FLAG-M2 antibody to detect induced FLAG protein. Lanes: 1. Precision Plus Protein Ladder (Bio-Rad), 2. Cytoplasmic control, 3. 0 mM NaCl wash, 4. 100 mM NaCl wash, 5. 200 mM NaCl wash, 6. 300 mM NaCl wash, 7. 400 mM NaCl wash, 8. 500 mM NaCl wash. *B,* Control blots of same samples using EZH2 antibody.

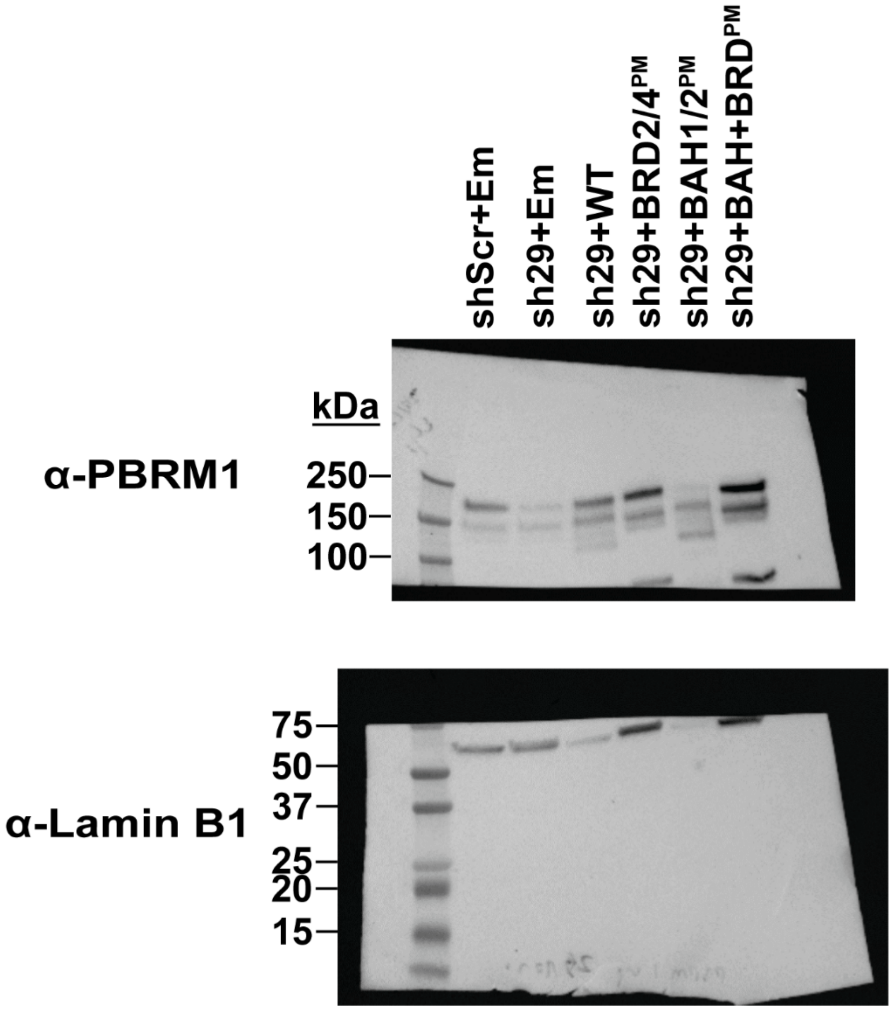

Figure S8. Western blots of total PBRM1 in HEK293T cells co-expressing knockdown and rescue PBRM1 vectors. Full western blots of whole cell extracts from HEK293T cells co-expressing shScr or shPBRM1 #29 along with indicated empty or full-length PBRM1 rescue vectors. Blots for PBRM1 (ABE70, Millipore) and Lamin B1 (abcam, ab16048) as a loading control are shown. Note: unexpectedly low levels of PBRM1 in BAH^{mut} rescue may be due to issues with sample preparation and/or epitope masking by the mutations in the BAH domains.
